# Engineering Cell Polarization Improves Protein Production in *Saccharomyces cerevisiae*

**DOI:** 10.3390/microorganisms10102005

**Published:** 2022-10-11

**Authors:** Shuo Yang, Junfeng Shen, Jiliang Deng, Hongxing Li, Jianzhi Zhao, Hongting Tang, Xiaoming Bao

**Affiliations:** 1 State Key Laboratory of Biobased Material and Green Papermaking, School of Bioengineering, Qilu University of Technology (Shandong Academy of Sciences), Jinan 250353, China; 2 Center for Synthetic Biochemistry, Shenzhen Institute of Synthetic Biology, Shenzhen Institutes for Advanced Technology, Chinese Academy of Sciences, Shenzhen 518055, China

**Keywords:** *Saccharomyces cerevisiae*, recombinant proteins, cell polarization, surface-display, secretion

## Abstract

*Saccharomyces cerevisiae* has been widely used as a microbial cell factory to produce recombinant proteins. Therefore, enhancing the protein production efficiency of yeast cell factories to expand the market demand for protein products is necessary. Recombinant proteins are often retained in the secretory pathway because of the limited protein transport performed by vesicle trafficking. Cell polarization describes the asymmetric organization of the plasma membrane cytoskeleton and organelles and tightly regulates vesicle trafficking for protein transport. Engineering vesicle trafficking has broadly been studied by the overexpression or deletion of key genes involved but not by modifying cell polarization. Here, we used α-amylase as a reporter protein, and its secretion and surface-display were first improved by promoter optimization. To study the effect of engineering cell polarization on protein production, fourteen genes related to cell polarization were overexpressed. *BUD1*, *CDC42*, *AXL1*, and *BUD10* overexpression increased the activity of surface-displayed α-amylase, and *BUD1*, *BUD3*, *BUD4*, *BUD7*, and *BUD10* overexpression enhanced secreted α-amylase activity. Furthermore, *BUD1* overexpression increased the surface-displayed and secreted α-amylase expression by 56% and 49%, respectively. We also observed that the combinatorial modification and regulation of gene expression improved α-amylase production in a dose-dependent manner. *BUD1* and *CDC42* co-overexpression increased the α-amylase surface display by 100%, and two genomic copies of *BUD1* improved α-amylase secretion by 92%. Furthermore, these modifications were used to improve the surface display and secretion of the recombinant β-glucosidase protein. Our study affords a novel insight for improving the surface display and secretion of recombinant proteins.

## 1. Introduction

In recent years, especially with the COVID-19 outbreak and the development of the biological industry, the market for biopharmaceutical proteins and industrial enzymes has reached hundreds of billions of dollars per year and continues to grow [1]. Further expansion of the market with high requirements has put pressure on the production capacity of protein products. Based on the production scale and cost, protein production in cell factories has become the best choice [2,3]. *Saccharomyces cerevisiae* has been widely used in protein production due to these advantages: (i) as a traditional eukaryotic model organism, it has a clear genetic background for genetical engineering; (ii) it has post−translational modification process as well as other eukaryotes, and thus, it can produce eukaryotic proteins with correct structure and high bioactivity [4]; and (iii) it has the capacity to withstand harsh environmental conditions [5].

In *S. cerevisiae*, recombinant proteins are often secreted extracellularly or displayed on the cell surface for diverse applications [6]. To continuously improve the protein expression capacity of *S. cerevisiae*, previous studies have focused on optimizing transcription levels, improving the protein synthesis process [7], and engineering secretory pathways [8,9,10,11,12]. Notably, engineering the key steps of the secretory pathway, including protein translocation, protein folding, glycosylation, and vesicle trafficking, has been extensively studied to improve the secretion and surface display of recombinant proteins. Recombinant proteins are often retained in the secretory pathway owing to the limitation of protein transport, and numerous efforts have been focused on improving vesicle trafficking to increase protein production. Vesicle transport is an essential step in the secretory pathway, which is responsible for transporting cargo proteins to their destination. In *S. cerevisiae*, recombinant protein overexpression resulted in intracellular accumulation [13] and enhanced vesicle transport from the endoplasmic reticulum (ER) to the Golgi and then to the cell membrane, which improved recombinant protein secretion and surface display [14], demonstrating that vesicle transport is a rate−limiting step in efficient protein expression.

Rapid vesicle transport is polarized, and secretory vesicles are transported along the actin cables to the budding sites [15]. Budding has two spatial patterns; the axial budding pattern that occurs in haploid cells and a bipolar budding pattern that occurs in diploid cells [16]. Currently, vesicle trafficking is mainly strengthened by the overexpression of key genes involved in vesicle budding, delivery, tethering, and fusion [14,17]. The axial budding pattern specifically requires four genes—*BUD3*, *BUD4*, *AXL1,* and *BUD10*—to maintain correct budding. Five genes—*BUD7*, *BUD8*, *BUD9*, *RAX1,* and *RAX2*—are involved in the bipolar budding pattern. However, *BUD1*, *BUD2*, and *BUD5* are required for both budding patterns. *S. cerevisiae* forms actin cables that connect the polar sites for budding during the cell cycle. During bud formation, membranes, lipids, and cell wall proteins are transported by secretory vesicles through actin cables to the cell membrane at the bud tip [18]. Secretory vesicle delivery depends on myosin Myo2p regulation associated with actin cables [19,20]. The loss of Myo2p activity no longer results in the transport of secretory vesicles [20]. The monomeric Rho-family GTPase Cdc42p is essential for establishing axial budding at the bud tip and polarizing the actin cytoskeleton [21]. Inactivation results in a defect in the fusion of secretory vesicles with the cell membrane at budding sites [22]. These results demonstrate that vesicle transport is tightly regulated by cell polarization in *S. cerevisiae.* Therefore, the identification of new gene targets involved in cell polarization is attractive for improving the production of recombinant proteins by regulating vesicle trafficking. However, engineering the cell polarization to enhance vesicle transport for efficient protein expression has rarely been reported.

In this study, α-amylase from *Aspergillus oryzae* was used as the reported protein and was expressed via secretion and surface display with promoter optimization. Genes involved in budding (*BUD3*, *BUD4*, *AXL1*, *BUD10*, *BUD7*, *BUD8*, *BUD9*, *RAX1*, *RAX2*, *BUD1*, *BUD2*, and *BUD5*), actin polarization (*CDC42*), and vesicle delivery (*MYO2*) were overexpressed to study the effect of engineered cell polarization on protein expression. Furthermore, the combinatorial modification of these genes was investigated. Finally, *Sacchromycopsis fibuligera* β-glucosidase (BGL1) was expressed in engineered strains to demonstrate the applicability of this strategy.

## 2. Materials and Methods

### 2.1. Strain and Media

The strains used in this study are listed in Table S1. The *Escherichia coli* strain Trans5α was used for the construction and propagation of plasmids. *E. coli* strains were cultivated in Luria−Bertani medium (5 g/L yeast extract, 10 g/L tryptone, and 10 g/L NaCl) with or without 100 μg/mL ampicillin at 37 °C for 12 h. *S. cerevisiae* strain Lab001 [23] and recombination strains were cultivated in YPD medium (10 g/L yeast extract, 20 g/L peptone, and 20 g/L glucose) or SC−AA medium [24] (190 mg/L arginine, 52 mg/L tyrosine, 108 mg/L methionine, 290 mg/L isoleucine, 440 mg/L lysine, 200 mg/L phenylalanine, 400 mg/L aspartic acid, 1260 mg/L glutamic acid, 380 mg/L valine, 220 mg/L threonine, 400 mg/L leucine, 130 mg/L glycine, 40 mg/L tryptophan, and 140 mg/L histidine) without uracil containing 20 g/L glucose at 30 °C, 200 rpm. *TPI1* is an essential gene in the glycolytic pathway commonly used as the selective marker to sustain high plasmid stability in rich medium and generate higher cell numbers and higher protein production. *TPI1*Δ strain does not grow on glucose as the sole carbon source and can grow on ethanol [25], and thus, was selected in SC/−URA Broth (Coolaber, Beijing, China) containing 20 g/L ethanol. Other deletion strains were selected in SC/−URA Broth containing 20 g/L glucose. After gene insertion, all gRNA plasmids were lost by 5−Fluoroorotic Acid (5−FOA) medium containing 8 g/L of SC/−URA Broth, 76 mg/L of uracil, 20 g/L of glucose, and 1 g/L of 5−FOA.

### 2.2. Plasmid and Strain Construction

The plasmids used in the present study are listed in Table S2. For *S. cerevisiae,* strain Lab001, derived from CEN.PK113−5D, was used as the background strain for all genetic manipulations. The promoters, terminators, homologous sequences, and other cell polarization genes were amplified from the Lab001 genome using the primers listed in Table S3. Construction of the expression cassettes and gene integration or deletion repairs were performed by fusion PCR. For the construction of the α-amylase−expressing plasmids, a high-copy plasmid with the *POT1* gene from *Schizosaccharomyces pombe*—a selection maker to complement the *TPI1* mutation [26]—was used as the backbone, and the α-amylase gene was amplified from pTH−Amys and inserted in the backbone, resulting in recombinant plasmid pCPOT [27]. The *TPI1Δ* strain containing pCPOT plasmid, therefore, allows for the stable expression in rich media (such as YPD) and has a high plasmid stability [25]. α-Amylase with or without the *SED1* sequence under the control of promoter *TPI1* or *TDH3* was inserted into pCPOT using the Gibson assembly method to form plasmids pCPOT1, pCPOT2, pCPOT3, and pCPOT4. To overexpress genes involved in cell polarization, a centromeric copy plasmid pPOT2 [28] with a UAR3 marker was used as the backbone, and the *TDH3* promoter, *CYC1* terminator, and genes of cell polarization were inserted into pPOT2 using the Gibson assembly method.

### 2.3. CRISPR−Cas9 Mediated Gene Insertion

The *S. cerevisiae* genome was modified using the CRISPR−Cas9 system. Gene deletion and expression cassette integration were performed using the CRISPR/Cas9 system, according to a previously described method [29]. All potential gRNAs for a target gene were produced using the Yeastriction web tool (http://yeastriction.tnw.tudelft.nl (accessed on 10 September 2021)). All gRNA plasmids were constructed following a previously described method and were verified by sequencing [30]. All native promoters, genes, homology sequences, and terminators were amplified using Lab001 genomic DNA as the template. Gene deletion or integration repairs were performed by fusion PCR using the primers listed in Table S3.

### 2.4. Enzymatic Assays

The strains expressing α-amylase or BGL1 were inoculated into YPD or SC−AA (2% glucose) medium and grown at 30 °C for 24 h. The fermentation broth was collected to measure total enzyme activity. The supernatant of the fermentation broth was collected by centrifugation to measure the extracellular activity. The collected cells were washed twice by PBS (pH 7.0) and suspended in PBS for measurement of intracellular activity. The α-amylase activity (Ceralpha Unit, CU) of the supernatant and the suspended cells was quantified and calculated by the same procedure according to the manufacturer’s instructions (Megazyme K−CERA, Wicklow, Ireland). The β-glucosidase activity was detected using *p*−nitrophenyl−β−d−glucopyranoside *p*NPG as the substrate, as described previously [26].

### 2.5. FACS

The following cultivation in YPD at 30 °C for 24 h, the cells were collected, washed twice with phosphate-buffered saline (PBS, pH 7.0), and suspended in PBS with 1 mg/mL bovine serum albumin (BSA) at a final OD_600_ of 1.0. Monoclonal mouse anti−V5−FITC antibody (Invitrogen−R96325) was added to the samples at 1:500 dilutions at 25 °C for 1 h. Cells were harvested and washed twice with PBS after immunostaining. The fluorescence distribution of 10,000 cells in each sample was analyzed using flow cytometry (CytoFLEX S, Beckman Coulter, Brea, CA, USA).

## 3. Results

### 3.1. Secretion and Surface Display of α-Amylase in S. cerevisiae

**Figure 1:**
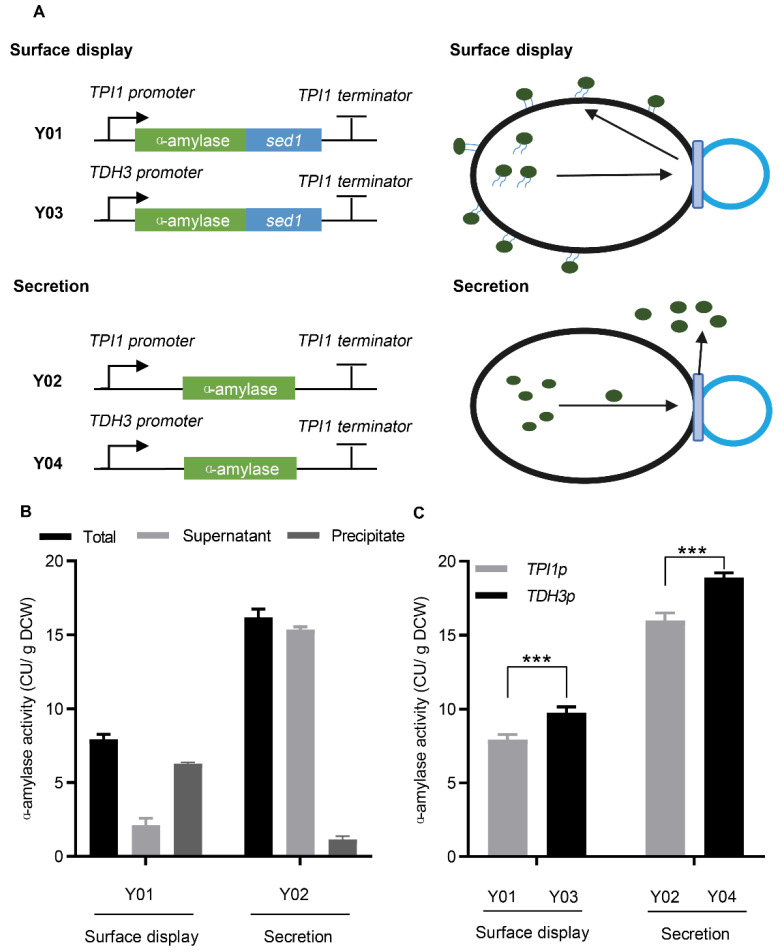
Expression of α-amylase in *Saccharomyces* *cerevisiae*. (**A**) Construction of α-amylase expression systems. (**B**) Activities of surface-displayed and secreted α-amylase. (**C**) The effect of promoters on the expression of α-amylase. All strains were cultivated in the YPD medium at 30 °C for 24 h. Data are mean ± SD from three independent experiments. The statistical significance of enzymatic assays was determined by a two−tailed homoscedastic (equal variance) *t*-test and three asterisks represents *p* < 0.005.

The α-amylase gene, with a signal peptide from *Kluyveromyces INU1*, was expressed for secretion under the control of the *TPI1* promoter. For surface display, the C−terminus of anchor protein, Sed1p, was fused downstream of α-amylase (Figure 1A). To determine the expression of α-amylase, the fermentation broth, including culture medium and cells, was collected for total α-amylase activity measurements. As shown in Figure 1B, α-amylase activity was detected in both secreting and surface-displaying strains, demonstrating that α-amylase was successfully expressed via secretion or surface display. Additionally, the activity of surface-displayed α-amylase was lower than that of secreted α-amylase, indicating that the fusion of the anchor protein may affect the expression of α-amylase. Secretion and surface display efficiency was analyzed by detecting the activity of α-amylase in the extracellular medium and cells. For surface-displayed α-amylase, the activity of cells was higher than that of the medium, whereas the results for secreted α-amylase were the opposite (Figure 1B). These results demonstrate that surface-displayed α-amylase was successfully anchored on the cell surface, even though a small amount escaped into the medium, whereas secreted α-amylase was mostly released into the medium.

To further improve α-amylase expression, we used the strong promoter, *TDH3p,* to increase the α-amylase transcription level. As shown in Figure 1C, the activity of surface-displayed and secreted α-amylase improved by 23% and 18%, respectively, when their expression was driven by the strong promoter, *TDH3p*. Additionally, eGFP expression under the *TDH3p* promoter was enhanced by 142% (Figure S1). The improvement in α-amylase expression was much lower than that in eGFP expression, demonstrating that the limiting step of α-amylase expression was not at the transcriptional level.

### 3.2. Cell Polarization Engineering Improved Protein Surface Display and Secretion

**Figure 2:**
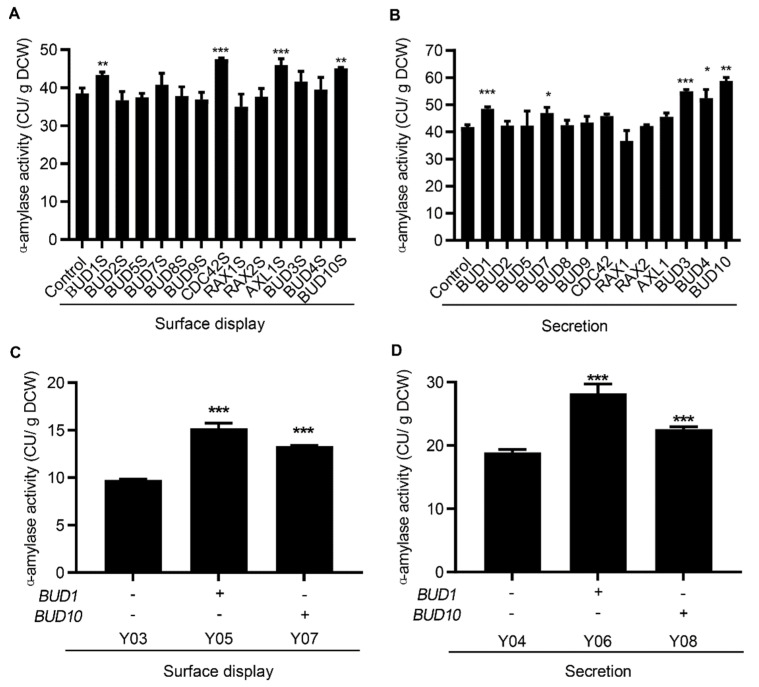
Engineering cell polarization enhanced α-amylase. The effect of engineering cell polarization on the activity of surface-displayed (**A**) and secreted (**B**) α-amylase. The effect of *BUD1* and *BUD10* expression on surface-displayed (**C**) and secreted (**D**) α-amylase. All strains involved in (**A**,**B**) were cultivated in the SC−AA medium without uracil at 30 °C for 24 h, and strains involved in (**C**,**D**) were cultivated in the YPD medium at 30 °C for 24 h. Data are mean ± SD from three independent experiments. The statistical significance of enzymatic assays was determined by a two−tailed homoscedastic (equal variance) *t*-test and one asterisk represents *p* < 0.05, two asterisks represent *p* < 0.01, and three asterisks represents *p* < 0.005.

Vesicle transport is a common rate−limiting step in the secretory pathway for efficient protein expression in *S. cerevisiae*. Rapid vesicle transport is regulated by cell polarization. Therefore, engineering the cell polarization to strengthen vesicle transport for efficient protein expression has been warranted. The *BUD3*, *BUD4*, *AXL1*, and *BUD10* genes are involved in axial budding; *BUD7*, *BUD8*, *BUD9*, *RAX1*, and *RAX2* are involved in bipolar budding; *BUD1, BUD2*, and *BUD5* are involved in both axial and bipolar budding; and the motor−encoding gene *MYO2* and GTPase−encoding gene *CDC42* were overexpressed by the centromeric plasmid. However, *MYO2* overexpression may be detrimental to cell health [31] and results in no recombinant strains. Compared to the expression in the control strain expressing an empty plasmid, *BUD1*, *CDC42*, *AXL1*, and *BUD10* overexpression increased the activity of surface-displayed α-amylase by 13%, 24%, 19%, and 17%, respectively (Figure 2A). As shown in Figure 2B, *BUD1*, *BUD3*, *BUD4, BUD7,* and *BUD10* overexpression enhanced the activity of secreted α-amylase by 16%, 32%, 25%, 12%, and 41%, respectively. These results suggested that the overexpression of *BUD1* and *BUD10* could simultaneously improve the surface display and secretion of α-amylase. *BUD1* and *BUD10* were expressed by integration into the genome to stabilize the gene expression. As shown in Figure 2C, compared with the control strain, *BUD1* and *BUD10* overexpression increased the activity of surface-displayed α-amylase by 56% and 37%, respectively. The secreted α-amylase activity improved by 49% and 20%, respectively (Figure 2D). In addition, the cell growth of these engineered strains was not affected (Figure S2A,B). These results demonstrate that the stable genomic expression of *BUD1* and *BUD10* facilitates the expression of α-amylase. Therefore, engineering cell polarization is beneficial for the expression of surface-displayed and secreted proteins.

### 3.3. Effect of Combinatorial Modifications on Protein Surface Display and Secretion

**Figure 3:**
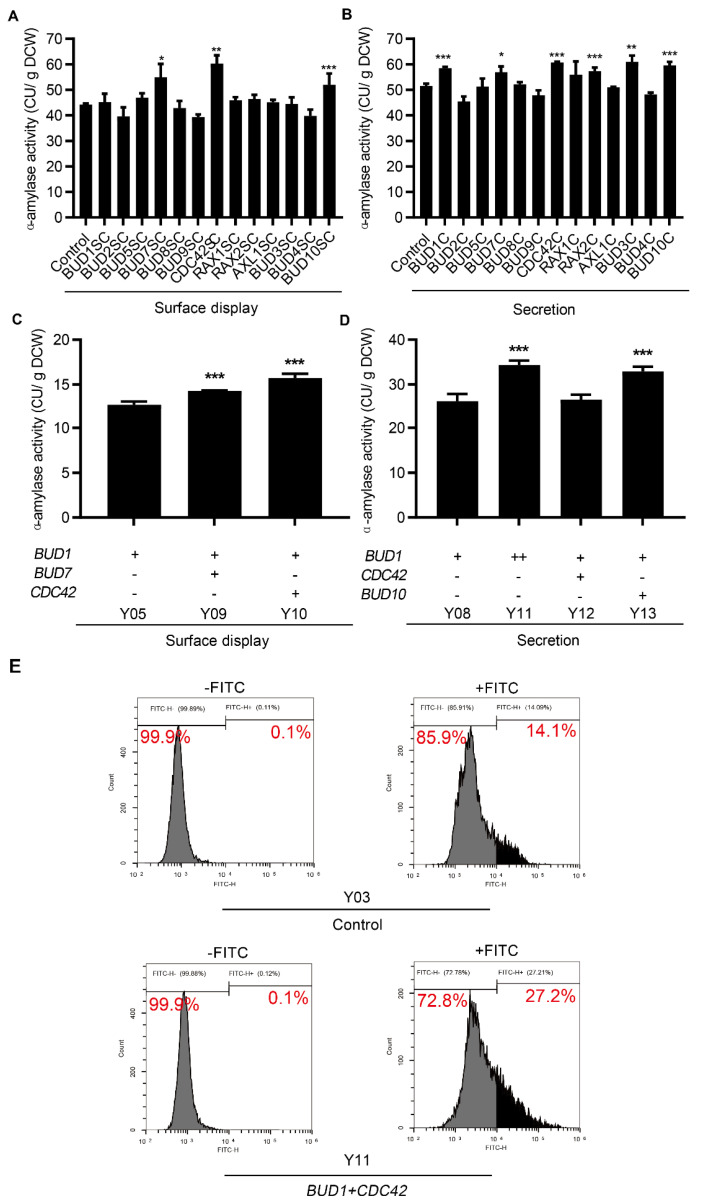
Effect of combinatorial modification of cell polarization on the production of α-amylase. The co-expression of *BUD1* with each component affected the activity of surface-displayed (**A**) and secreted (**B**) α-amylase. The effect of genomic co-expression on surface-displayed (**C**) and secreted (**D**) α-amylase. (**E**) FACS analysis of surface-displayed α-amylase. All strains involved in (**A**) and (**B**) were cultivated in the SC−AA medium without uracil at 30 °C for 24 h, and strains involved in (**C**,**D**) were cultivated in the YPD medium at 30 °C for 24 h. Data are mean ± SD from three independent experiments. The statistical significance of enzymatic assays was determined by a two−tailed homoscedastic (equal variance) *t*-test and one asterisk represents *p* < 0.05, two asterisks represent *p* < 0.01, and three asterisks represents *p* < 0.005. (+, overexpressing one copy of gene; ++, overexpressing two copies of gene).

The effect of cell polarization on protein expression may be regulated by the coordination between multiple genes involved in cell polarization. Therefore, combinatorial gene modifications were further explored to study their effects on protein production. We overexpressed polarization−related genes using a centromeric plasmid in the Y05 and Y06 strains, which improved surface-displayed and secreted α-amylase via the overexpression of one copy of the *BUD1* gene. As shown in Figure 3A, the co-overexpression of *BUD7*, *CDC42*, and *BUD10* with *BUD1* further increased the activity of surface-displayed α-amylase by 24%, 36%, and 17%, respectively, compared to the control strain, which expressed an empty plasmid. The effect of cell polarization on protein expression may be regulated by coordination between multiple genes involved in cell polarization. Therefore, combinatorial gene modifications were further explored to study their effects on protein production. We overexpressed polarization−related genes using a centromeric plasmid in the Y05 and Y06 strains, which improved surface-displayed and secreted α-amylase via the overexpression of one copy of the *BUD1* gene. As shown in Figure 3A, the co-overexpression of *BUD7*, *CDC42*, and *BUD10* with *BUD1* further increased the activity of surface-displayed α-amylase by 24, 36, and 17%, respectively, compared to the control strain, which expressed an empty plasmid. The activity of secreted α-amylase increased by 11%, 18%, 11%, 18%, and 12% when *BUD7*, *CDC42*, *RAX2*, *BUD3*, and *BUD10* were overexpressed with *BUD1*, respectively (Figure 3B). In addition, a further increase in *BUD1* expression enhanced the activity of secreted α-amylase by 14%, but not surface-displayed α-amylase. These results showed that the co-overexpression of *BUD7*, *CDC42*, and *BUD10* with *BUD1* could simultaneously improve the surface display and secretion of α-amylase. The expression of *BUD7* and *CDC42* notably improved the expression of surface-displayed α-amylase, and *CDC42* and *BUD10* had the best performance on secreted α-amylase expression. They were then inserted into the Y05 and Y06 genomes to construct strains with stable expression. We also increased the copy number of genomic *BUD1* in the α-amylase−secreting strain. As shown in Figure 3C, the activity of surface-displayed α-amylase improved by 14% and 24% with the genomic overexpression of *BUD7* and *CDC42*, respectively. The genomic overexpression of *BUD10* improved the activity of secreted α-amylase by 25%, whereas the *CDC42* expression did not further enhance the α-amylase expression, which may be caused by the low expression of genomic *CDC42* compared to plasmid expression (Figure 3D). Extra−genomic *BUD1* expression further increased the expression of secreted α-amylase by 31%. The OD_600_ of these engineered strains was not affected (Figure S3C,D). However, the growth rate of Y11 expressing two copies of *BUD1* gene reduced slightly (Figure S4A,B). These results revealed that the co-expression of genes involved in cell polarization synergistically improved the protein expression.

The α-amylase activity of the cells and culture medium was determined to evaluate the efficiency of surface display and secretion in the engineered strains. As shown in Figure S3A, most of the α-amylase activity was detected in the cells. Fluorescence−activated cell sorting (FACS) analysis was performed to analyze the expression of surface-displayed α-amylase using antibody−labeled FITC to recognize the C-terminal V5 tag of α-amylase. In the control strain without cell polarization engineering, 14.09% of cells had fluorescent signals in the presence of the antibody, and no stained cells were detected in the absence of the antibody (Figure 3E). The *BUD1* and *CDC42* overexpressing strains exhibited that 27.21% of the population were positively stained by the addition of antibodies, whereas they had no detectable fluorescent signals without an antibody addition (Figure 3E). In comparison, in the α-amylase−secreting strain, a large part of α-amylase activity, was distributed in the extracellular medium (Figure S3B). These results indicate that engineering cell polarization improved the surface display and secretion of α-amylase.

### 3.4. Applicability Testing of Engineering Cell Polarization

**Figure 4:**
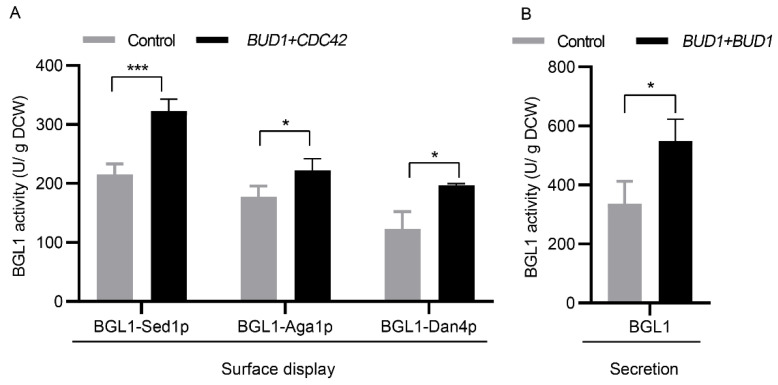
Engineering cell polarization enhanced the surface-displayed (**A**) and secretion (**B**) of BGL1. All strains were cultivated in the SC−AA medium without uracil at 30 °C for 24 h. Data are mean ± SD from three independent experiments. The statistical significance of enzymatic assays was determined by a two−tailed homoscedastic (equal variance) *t*-test and one asterisk represents *p* < 0.05, and three asterisks represents *p* < 0.005.

β-glucosidase (BGL1) from *Saccharomycopsis fibuligera* was used as an additional reporter to evaluate whether engineering cell polarization has an overall positive effect on the surface display and secretion of proteins because this enzyme plays an essential role in the production of cellulosic chemicals. We expressed surface-displayed BGL1 with different anchor proteins, including Sed1p, Aga1p, and Dan4p, using high−copy plasmids in strains with or without genomic *BUD1* and *CDC42* overexpression. The results showed that the co-expression of *BUD1* and *CDC42* increased the activity of BGL1 with Sed1p, Aga1p, and Dan4p by 50%, 25%, and 60%, respectively (Figure 4A). Secreted BGL1 was also expressed, and the activity was increased by 50% when two copies of genomic *BUD1* were expressed (Figure 4B). Therefore, cell polarization modification can be used as a general strategy to improve the surface display and secretion of recombinant proteins in *S. cerevisiae*.

## 4. Discussion

*BUD1* and *BUD10* overexpression increases the activity of both surface-displayed and secreted α-amylase. However, the overexpression of *CDC42* and *AXL1* only improved the activity of surface-displayed α-amylase but not secreted α-amylase. In contrast, the overexpression of *BUD3* and *BUD4* increased the activity of secreted α-amylase but not surface-displayed α-amylase. The results indicated that secreted and surface-displayed proteins showed different restrictions in regulating cell polarization. Previous studies have reported that the cargo receptor/adaptor in vesicle trafficking from the ER to the Golgi required by surface-displayed proteins that contain the GPI domain is different from that of secreted proteins [32,33,34]. Surface-displayed and secreted proteins are also transported by different vesicles during protein transport from the Golgi to the cell membrane [35,36,37]. Therefore, the difference in engineered cell polarization may be caused by the different properties of vesicle trafficking of surface-displayed proteins.

The co-overexpression of *BUD1* with *BUD7* and *CDC42* further increased the surface expression of α-amylase. *BUD1* expression in the presence of *BUD10* further improved α-amylase secretion. Additionally, we found that an increased *BUD1* expression enhanced the activity of secreted α-amylase. Furthermore, these modifications can improve surface display using different anchor proteins and secretion of BGL1. These results indicate that combinatorial modifications and regulation of the expression dose of cell polarization components had a better performance in the production of recombinant proteins in *S. cerevisiae*. In the future, these new genes could be used as candidates for engineering with previously identified targets and for having synergistic effects, such as *SRP54* and *SRP14* involved in protein translocation [27], *PDI1* and *KAR2* [38,39] involved in protein folding, *OCH1* and *MNN9* involved in glycosylation [40], *SSO1* and *SNC2* [14] involved in vesicle trafficking, and *VPS10*, *YAP3*, and *PEP4* involved in protein degradation [41,42,43].

In conclusion, our study identified several targets involved in cell polarization to improve protein surface display and secretion. The results demonstrated that the expression of surface-displayed and secreted α-amylase was not limited by transcriptional levels and suggested that the overexpression of cell polarization components using plasmids increased the activity of surface-displayed and secreted α-amylase. We also found that the co-overexpression of these gene targets had synergistic effects on α-amylase activity. In addition, the regulation of gene expression affects α-amylase production. Furthermore, we constructed stable strains by integrating functional genes into the genome and applied them to improve the surface display and secretion of another recombinant protein, β-glucosidase. Our strategy can effectively improve the application of surface-displayed recombinant protein in environmental protection [44], bio−catalysis [45], drug screening [46], library screening [47] and secreted recombinant protein in bio-energy [26] and biopharmaceuticals [48]. Collectively, this study provides a general strategy for the design and construction of microbial cell factories to improve the production of surface-displayed or secreted recombinant proteins in yeast.

## Data Availability

Not applicable.

## Supplementary Materials

The following supporting information can be downloaded at: https://www.mdpi.com/article/10.3390/microorganisms10102005/s1, Figure S1: The expression of eGFP driven by *TPI1p* and *TDH3p*; Figure S2: Cell growth of surface-displaying (A) and secreting (B) strains with the genomic expression of *BUD1* or *BUD10*; Figure S3: The effect of combinatorial modifications on the production of α-amylase and cell growth; Figure S4: The growth curve (A) and the maximum specific growth rate of engineered strains. Table S1: Strains used in this study [23]; Table S2: Plasmids used in this study [26,27,28,49]; Table S3: Primers used in this study.
