# Supplementary material for: Engineering Cell Polarization Improves Protein Production in Saccharomyces cerevisiae
Source: Microorganisms. 2022 Oct 11;10(10):2005. doi: 10.3390/microorganisms10102005 (PMC9609600; doi:10.3390/microorganisms10102005)
Supplement: Supplementary file 1 [file microorganisms-10-02005-s001.zip › microorganisms-1957712-supplementary.pdf]

Supporting information

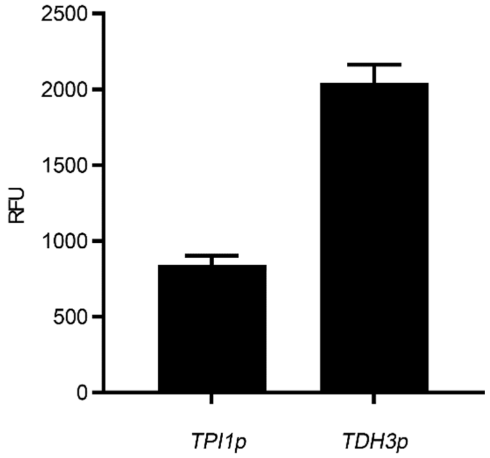

Figure **S1.** Expression of eGFP driven by *TPI1p* and *TDH3p*. The eGFP was expressed in Lab001 by centromeric plasmid driven by TPI1p and TDH3p. All strains were cultivated in the YPD medium at 30 °C for 24 h. Data are mean ± SD from three independent experiments. RFU: relative fluorescence units, fluorescence intensity per unit of OD_{600}.

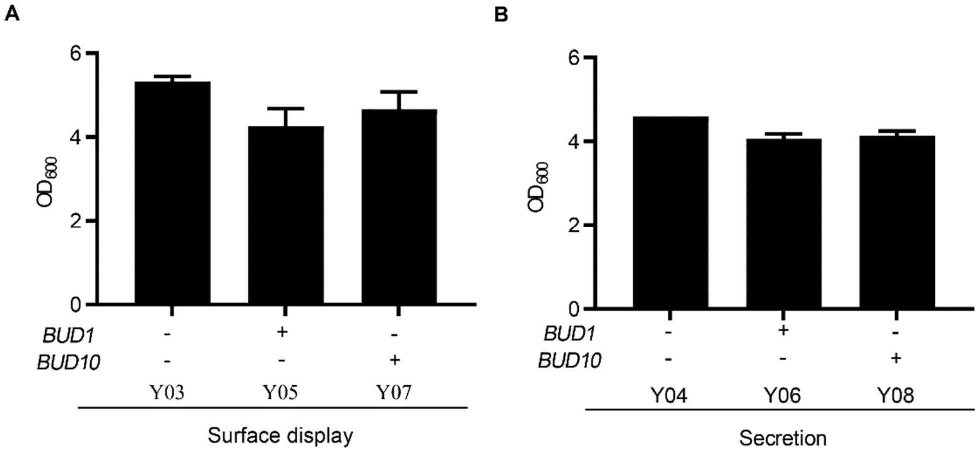

Figure **S2**. Cell growth of surface−displaying (A) and secreting (B) strains with the genomic expression of *BUD1* or *BUD10*. All strains were cultivated in the YPD medium at 30 °C for 24 h. Data are mean ± SD from three independent experiments.

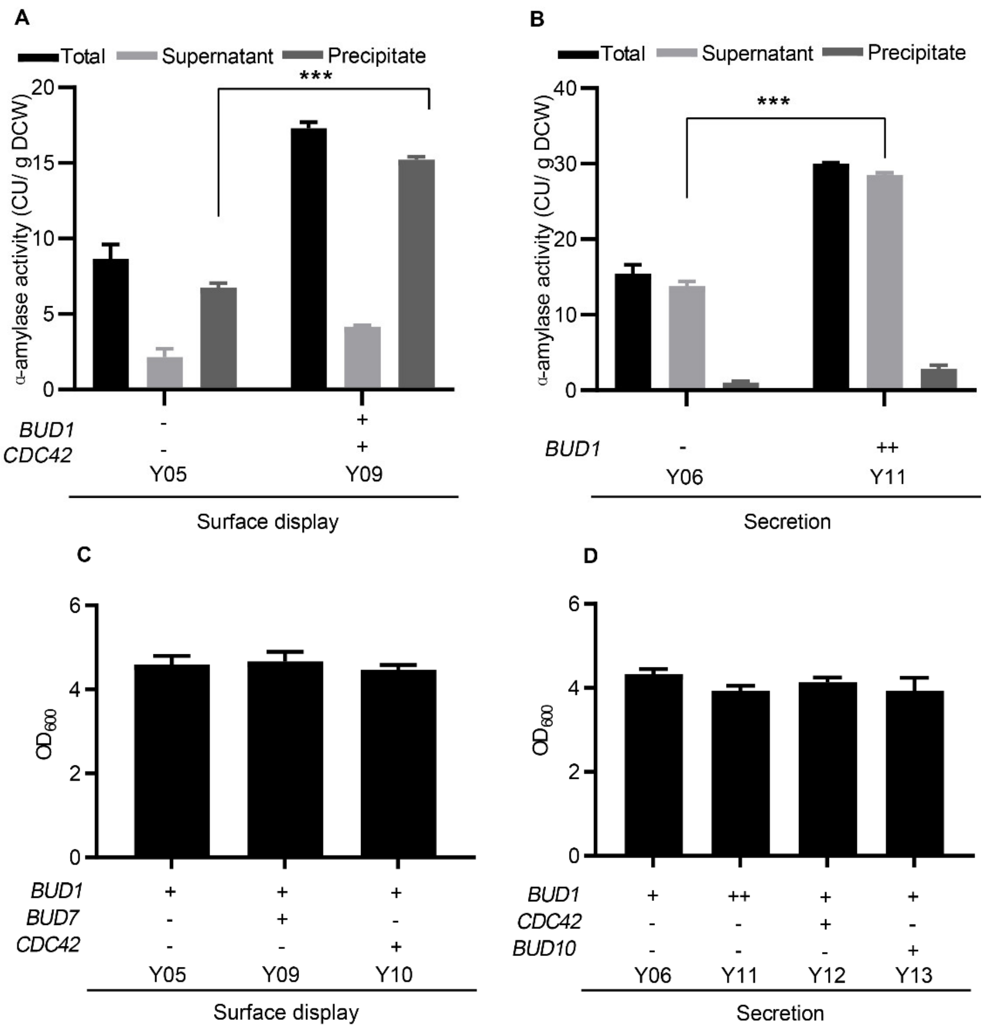

Figure S3. The effect of combinatorial modifications on the production of α−amylase and cell growth. (A) Combinatorial modifications increased the surface display of α−amylase. (B) Combinatorial modifications increased the secretion of α−amylase. The cell growth of combinatorial modifications in surface-displaying (C) and secreting (D) strains. All strains were cultivated in the YPD medium at 30 °C for 24 h. Data are mean ± SD from three independent experiments. The statistical significance of enzymatic assays was determined by a two−tailed homoscedastic (equal variance) t−test and three asterisks represents P < 0.005.

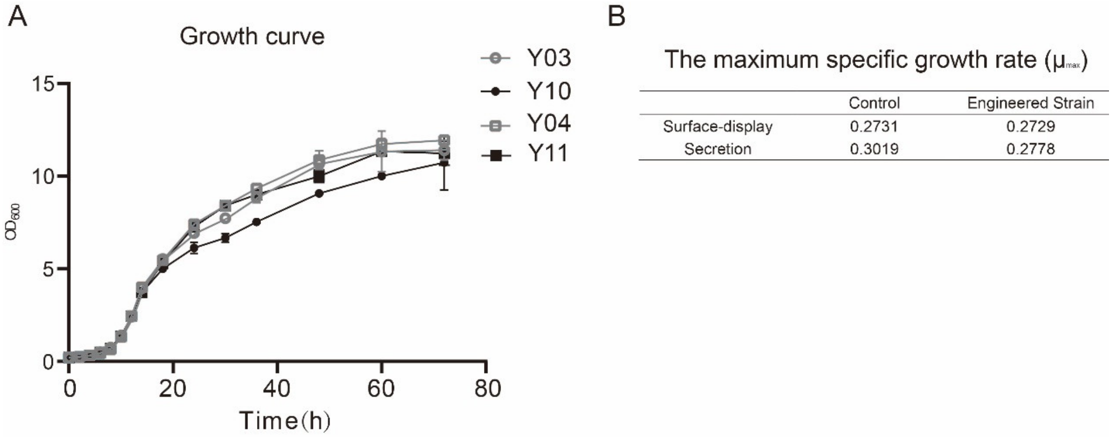

Figure S4. The growth curve (A) and the maximum specific growth rate of engineered strains. All strains were cultivated in the YPD medium at 30 °C for 72 h. Data are mean ± SD from three independent experiments.

Table S1. Strains used in this study

Strains	Relevant characteristics/ genotype	Reference
Lab001	MATa ura3-52 can1Δ::cas9-natNT2 TRP1 LEU2 HIS3	[1]
Y01	Lab001 Δtpi; pCPOT1	This study
Y02	Lab001 Δtpi; pCPOT2	This study
Y03	Lab001 Δtpi; pCPOT3	This study
Y04	Lab001 Δtpi; pCPOT4	This study
Y05	Lab001 Δtpi; X1-1::pTDH3-BUD1-tCYC1; pCPOT3	This study
Y06	Lab001 Δtpi; X1-1::pTDH3-BUD1-tCYC1; pCPOT4	This study
Y07	Lab001 Δtpi; XI1-5::pTDH3-BUD10-tCYC1; pCPOT3	This study
Y08	Lab001 Δtpi; XI1-5::pTDH3-BUD10-tCYC1; pCPOT4	This study
Y09	Lab001 Δtpi; X1-1::pTDH3-BUD1-tCYC1; XII-2::pTDH3-BUD7-tCYC1 pCPOT3	This study
Y10	Lab001 Δtpi; X1-1::pTDH3-BUD1-tCYC1; XII-2::pTDH3-CDC42-tCYC1 pCPOT3	This study
Y11	Lab001 Δtpi; X1-1::pTDH3-BUD1-tCYC1; XII-2::pTDH3-BUD1-tCYC1 pCPOT4	This study
Y12	Lab001 Δtpi; X1-1::pTDH3-BUD1-tCYC1; XII-2::pTDH3-CDC42-tCYC1 pCPOT4	This study
Y13	Lab001 Δtpi; X1-1::pTDH3-BUD1-tCYC1; XII-2::pTDH3-BUD10-tCYC1 pCPOT4	This study
BUD1S	Y03;pBUD1	This study
BUD2S	Y03;pBUD2	This study
BUD5S	Y03;pBUD5	This study
BUD7S	Y03;pBUD7	This study
BUD8S	Y03;pBUD8	This study
BUD9S	Y03;pBUD9	This study
CDC4S	Y03;pCDC42	This study

RAX1S	Y03;pRAX1	This study
RAX2S	Y03;pRAX2	This study
AXL1S	Y03;pAXL1	This study
BUD3S	Y03;pBUD3	This study
BUD4S	Y03;pBUD4	This study
BUD10S	Y03;pBUD10	This study
BUD1	Y04;pBUD1	This study
BUD2	Y04;pBUD2	This study
BUD5	Y04;pBUD5	This study
BUD7	Y04;pBUD7	This study
BUD8	Y04;pBUD8	This study
BUD9	Y04;pBUD9	This study
CDC42	Y04;pCDC42	This study
RAX1	Y04;pRAX1	This study
RAX2	Y04;pRAX2	This study
AXL1	Y04;pAXL1	This study
BUD3	Y04;pBUD3	This study
BUD4	Y04;pBUD4	This study
BUD10	Y04;pBUD10	This study
BUD1SC	Y05;pBUD1	This study
BUD2SC	Y05;pBUD2	This study
BUD5SC	Y05;pBUD5	This study
BUD7SC	Y05;pBUD7	This study
BUD8SC	Y05;pBUD8	This study
BUD9SC	Y05;pBUD9	This study
CDC42SC	Y05;pCDC42	This study
RAX1SC	Y05;pRAX1	This study
RAX2SC	Y05;pRAX2	This study
AXL1SC	Y05;pAXL1	This study
BUD3SC	Y05;pBUD3	This study
BUD4SC	Y05;pBUD4	This study
BUD10SC	Y05;pBUD10	This study
BUD1C	Y06;pBUD1	This study
BUD2C	Y06;pBUD2	This study
BUD5C	Y06;pBUD5	This study
BUD7C	Y06;pBUD7	This study
BUD8C	Y06;pBUD8	This study
BUD9C	Y06;pBUD9	This study
CDC42C	Y06;pCDC42	This study
RAX1C	Y06;pRAX1	This study
RAX2C	Y06;pRAX2	This study
AXL1C	Y06;pAXL1	This study
BUD3C	Y06;pBUD3	This study

BUD4C	Y06;pBUD4	This study
BUD10C	Y06;pBUD10	This study
BGL1	Y11; pJBGL1	This study
BGL1-SED1p	Y10; pJBGL-L-SED1	This study
BGL1-AGA1p	Y10; pJBGL-L-AGA1	This study
BGL1-DAN4p	Y10; pJBGL-L-DAN4	This study

Table S2. Plasmids used in this study

Strains	Relevant characteristics/ genotype	Reference
pCPOT	Yeast 2μ plasmid with POT1 gene from S. pombe as a selection marker	[2]
pCPOT1	pCPOT with TPI1 promoter, TPI1 terminator and INU1-a-amylase-V5-linker-SED1	This study
pCPOT2	pCPOT with TPI1 promoter, TPI1 terminator and INU1-a-amylase-V5	This study
pCPOT3	pCPOT with TDH3 promoter, TPI1 terminator and INU1-a-amylase-V5-linker-SED1	This study
pCPOT4	pCPOT with TDH3 promoter, TPI1 terminator and INU1-a-amylase-V5	This study
pPOT2	Yeast centromeric plasmid with URA3 marker	Laboratory
pTDH3	pPOT2 with TDH3 promoter, ADH1 terminator and eGFP	[3]
pTPI1	pPOT2 with TPI1 promoter, ADH1 terminator and eGFP	[3]
pBUD1	pPOT2 with TDH3 promoter, CYC1 terminator and BUD1 gene	This study
pBUD2	pPOT2 with TDH3 promoter, CYC1 terminator and BUD2 gene	This study
pBUD5	pPOT2 with TDH3 promoter, CYC1 terminator and BUD5 gene	This study
pBUD7	pPOT2 with TDH3 promoter, CYC1 terminator and BUD7 gene	This study
pBUD8	pPOT2 with TDH3 promoter, CYC1 terminator and BUD8 gene	This study
pBUD9	pPOT2 with TDH3 promoter, CYC1 terminator and BUD9 gene	This study
pCDC42	pPOT2 with TDH3 promoter, CYC1 terminator and CDC42 gene	This study
pRAX1	pPOT2 with TDH3 promoter, CYC1 terminator and RAX1 gene	This study
pRAX2	pPOT2 with TDH3 promoter, CYC1 terminator and RAX2 gene	This study

pAXL1	pPOT2 with TDH3 promoter, CYC1 terminator and AXL1 gene	This study
pBUD3	pPOT2 with TDH3 promoter, CYC1 terminator and BUD3 gene	This study
pBUD4	pPOT2 with TDH3 promoter, CYC1 terminator and BUD4 gene	This study
pBUD10	pPOT2 with TDH3 promoter, CYC1 terminator and BUD10 gene	This study
pJFE3	Yeast 2μ plasmid with URA3 marker	[2]
pJBGL1	pJFE3 with TEF1 promoter, PGK1 terminator and BGL1-linker-V5 tag	Laboratory
pJBGL-L-AGA1	pJFE3 with TEF1 promoter, PGK1 terminator and BGL1-linker-V5-AGA1	[4]
pJBGL-L-DAN4	pJFE3 with TEF1 promoter, PGK1 terminator and BGL1-linker-V5-DAN4	[4]
pJBGL-L-SED1	pJFE3 with TEF1 promoter, PGK1 terminator and BGL1-linker-V5-SED1	[4]
pQC030	pMEL10 with gRNA of XI-1	Laboratory
pQC007	pMEL10 with gRNA of XII-2	Laboratory
pTH-amys	Yeast centromeric plasmid with HIS marker, PGK1 promoter, CYC1 terminator and α-amylase gene from A. oryzae with SUC2 signal peptide	[5]
pQCTPI1	pMEL10 with gRNA of TPI1	This study

Table S3. Primers used in this study

Primer	Sequence 5′→3′
backbone-pPOT2-f	TTTGGGACGCTCGAAGGCTTTAATTTGCGGCCGGTACCCCCTCAGGCAGAGACCCAAGA
backbone-pPOT2-r	GTATTCTTTGAAATGGCAGTATTGATAATGATAAACTCGAAGCCAGGTAGAGACCGAGT
TDH3p-f	TCGAGTTTATCATTATCAATACTGCCATTTC
TDH3p-r	CATTTTGTTTGTTTATGTGTGTTTATTCGAAACTAA
CYC1t-f	GATACCGTCGACCTCGAGTC
CYC1t-r	GGGTACCGGCCGCAAATTAAAG
Bud1-f	TCGAATAAACACACATAAACAAACAAAATGAGAGACTATAAATTAGTAGTATTGGGTGC
Bud1-r	ATAACTAATTACATGACTCGAGGTCGACGGTATCCTATAGAATAGTGCAAGTGGAAGCG
BUD2-f	GAACTTAGTTTCGAATAAACACACATAAACAAACAAAATGAGCTCCAACAATGAACCGG
BUD2-r	AATTACATGACTCGAGGTCGACGGTATCTTAAGATCCCCCTGTTTCTTTTTCTTTTTG

BUD5-f	GAATAAACACACATAAACAAACAAAATGAGCCCGAAAAACAAATATGTATATATCTGTG
BUD5-r	CATAACTAATTACATGACTCGAGGTCGACGGTATCTTAGGTAAGCCTTGGAACCTTAGC
BUD7-f	AGTTTCGAATAAACACACATAAACAAACAAAATGATTACGCAGAATTCTATACCAGAGG
BUD7-r	CATAACTAATTACATGACTCGAGGTCGACGGTATCTTATGCATCGTAGCATCCAATGGT
BUD8-f	TCGAATAAACACACATAAACAAACAAAATGATACAATCAGACGAAGATAATTTGGACTC
BUD8-r	CTAATTACATGACTCGAGGTCGACGGTATCTTATTCACGTGTTATACCCACACCAAATC
BUD9-f	TCGAATAAACACACATAAACAAACAAAATGACGAAAATAACCAGAGATGTTTCGATAAC
BUD9-r	TAATTACATGACTCGAGGTCGACGGTATCTTACTCTCTTATTATTCCCACACCAAATCC
CDC42-f	CAAGAACTTAGTTTCGAATAAACACACATAAACAAACAAAATGCAAACGCTAAAGTGTG
CDC42-r	ACTAATTACATGACTCGAGGTCGACGGTATCCTACAAAATTGCACATTTTTTACTTTTC
RAX1-f	TTAGTTTCGAATAAACACACATAAACAAACAAAATGAAGGAAGAGCTCAGCAAAGTGAG
RAX1-r	GTGACATAACTAATTACATGACTCGAGGTCGACGGTATCTCATACACGACGGCCGGGAA
RAX2-f	CTTAGTTTCGAATAAACACACATAAACAAACAAAATGTTTGTTCATCGTCTCTGGACAC
RAX2-r	TAATTACATGACTCGAGGTCGACGGTATCTTAGACAAACTTCATAAGTTTTTCGGGTGG
AXL1-f	TTCGAATAAACACACATAAACAAACAAAATGTCCTTGAGAGAAGTAACTAATTATGAAG
AXL1-r	GCGTGACATAACTAATTACATGACTCGAGGTCGACGGTATCTCAAAATGCGGCCTTGCG
BUD3-f	GAACTTAGTTTCGAATAAACACACATAAACAAACAAAATGGAGAAAGACCTGTCGTCTC
BUD3-r	CTAATTACATGACTCGAGGTCGACGGTATCTCAATAAAATTTCAGTTTCTCTGGGCCAC
BUD4-f	AAGAACTTAGTTTCGAATAAACACACATAAACAAACAAAATGCACGACGCAGAGAGTAC
BUD4-r	ATTACATGACTCGAGGTCGACGGTATCCTAATTAAAATCTTGCTTCAAATTATGACCGG
BUD10-f	AGTTTCGAATAAACACACATAAACAAACAAAATGACACAGCTTCAGATTTCATTATTGC
BUD10-r	TGACATAACTAATTACATGACTCGAGGTCGACGGTATCTCACAGCATTTCTGGGATGCG

MYO2-f	CTTAGTTTCGAATAAACACACATAAACAAACAAAATGTCTT TTGAAGTGGGTACACGAT
MYO2-r	TGACATAACTAATTACATGACTCGAGGTCGACGGTATCTTA GTGGCCGTCTTGAACGAC
XI-1 up fw	ATTTGTGTGAAGGAATAGTGACG
XI-1 up rv	GTATTCTTTGAAATGGCAGTATTGATAATGATAAACTCGAC AATGGGCTTGGTATTCCG
XI-1 down fw	TTTGGGACGCTCGAAGGCTTTAATTTGCGGCCGGTACCCTTT CTTGGCATTGGCAAATC
XI-1 down rv	AAGAGCCGAGTCCCCATCAG
XII-2 up fw	CGGCATGCAAACATCTACAC
XII-2 up rv	TATTCTTTGAAATGGCAGTATTGATAATGATAAACTCGACAT AACGCGTTACACGGAAG
XII-2 down fw	TTGGGACGCTCGAAGGCTTTAATTTGCGGCCGGTACCCCTAC TATCGGCGACTCTCTCG
XII-2 down rv	GAGCGAACGTAAGAGAGGTTAATG
XI-1 det fw	CTTAATGGGTAGTGCTTGACACG
XI-1 det rv	GAAGACCCATGGTTCCAAGGA
XII-2 det fw	CGAAGAAGGCCTGCAATTC
XII-2 det rv	GGCCCTGATAAGGTTGTTG
CPOT-backobone-r	GGTACCTTTTAGTTTATGTATGTG
CPOT-backobone-f	TAACTCGAGTCTAGAAACTAAG
amy-f	CACATACATAAACTAAAAGGTACCATGAAGTTCGCATACTC CCTC
amy-sed-r	CTTAGTTTCTAGACTCGAGTTATAAGAATAACATAGCAACA CC
amy-r	CTTAGTTTCTAGACTCGAGTTAAGTAGAATCTAAACCCAAC AAAGG
CPOT-r2	GGCCGGCCGGATCCATGCCAACGGTTG
CPOT-f2	GGTACCATGAAGTTCGCATACTCCC
amy-pTDH3-f	TGGCATGGATCCGGCCGGCCTCATTATCAATACTGCCATTTC
amy-pTDH3-r	TATGCGAACTTCATGGTACCTTTGTTTGTTTATGTGTGTTTAT TCG

References

1. Zheng, T.; Zhang, M.; Wu, L.; Guo, S.; Liu, X.; Zhao, J.; Xue, W.; Li, J.; Liu, C.; Li, X.; et al. Upcycling CO2 into energy-rich long-chain compounds via electrochemical and metabolic engineering. *Nature Catalysis* **2022**, *5*, 388-396, doi:10.1038/s41929-022-00775-6.
2. Tang, H.; Hou, J.; Shen, Y.; Xu, L.; Yang, H.; Fang, X.; Bao, X. High β-glucosidase secretion in *Saccharomyces cerevisiae* improves the efficiency of cellulase hydrolysis and ethanol production in simultaneous saccharification and fermentation. *J Microbiol Biotechnol* **2013**, *23*, 1577-1585, doi:10.4014/jmb.1305.05011.

3. Deng, J.; Wu, Y.; Zheng, Z.; Chen, N.; Luo, X.; Tang, H.; Keasling, J.D. A synthetic promoter system for well-controlled protein expression with different carbon sources in *Saccharomyces cerevisiae*. *Microb Cell Fact* **2021**, *20*, 202, doi:10.1186/s12934-021-01691-3.
4. Yang, X.; Tang, H.; Song, M.; Shen, Y.; Hou, J.; Bao, X. Development of novel surface display platforms for anchoring heterologous proteins in *Saccharomyces cerevisiae*. *Microb Cell Fact* **2019**, *18*, 85, doi:10.1186/s12934-019-1133-x.
5. Tang, H.; Bao, X.; Shen, Y.; Song, M.; Wang, S.; Wang, C.; Hou, J. Engineering protein folding and translocation improves heterologous protein secretion in *Saccharomyces cerevisiae*. *Biotechnol Bioeng* **2015**, *112*, 1872-1882, doi:10.1002/bit.25596.
